# Role of flavonoids in controlling obesity: molecular targets and mechanisms

**DOI:** 10.3389/fnut.2023.1177897

**Published:** 2023-05-12

**Authors:** Anns Mahboob, Samson Mathews Samuel, Arif Mohamed, Mohmmad Younus Wani, Sofiane Ghorbel, Nabil Miled, Dietrich Büsselberg, Ali Chaari

**Affiliations:** ^1^Department of Pre-medical Education, Weill Cornell Medicine-Qatar, Education City, Qatar Foundation, Doha, Qatar; ^2^Department of Physiology and Biophysics, Weill Cornell Medicine-Qatar, Education City, Qatar Foundation, Doha, Qatar; ^3^College of Science, University of Jeddah, Jeddah, Saudi Arabia; ^4^Science and Arts at Khulis, University of Jeddah, Jeddah, Saudi Arabia

**Keywords:** anti-obesity therapy, flavonoids, metabolic syndrome, natural compounds, obesity

## Abstract

Obesity presents a major health challenge that increases the risk of several non-communicable illnesses, such as but not limited to diabetes, hypertension, cardiovascular diseases, musculoskeletal and neurological disorders, sleep disorders, and cancers. Accounting for nearly 8% of global deaths (4.7 million) in 2017, obesity leads to diminishing quality of life and a higher premature mortality rate among affected individuals. Although essentially dubbed as a modifiable and preventable health concern, prevention, and treatment strategies against obesity, such as calorie intake restriction and increasing calorie burning, have gained little long-term success. In this manuscript, we detail the pathophysiology of obesity as a multifactorial, oxidative stress-dependent inflammatory disease. Current anti-obesity treatment strategies, and the effect of flavonoid-based therapeutic interventions on digestion and absorption, macronutrient metabolism, inflammation and oxidative stress and gut microbiota has been evaluated. The use of several naturally occurring flavonoids to prevent and treat obesity with a long-term efficacy, is also described.

## 1. Background

The epidemic of obesity (the abnormal or excessive accumulation of fat in the body) has tripled over the past 5 decades among adults and children (1, 2). As a complex multifactorial disease, obesity is significantly detrimental to the health of the affected individuals and increases the risk of several comorbid conditions (3). Obesity and the diseases associated with it are thus a growing concern among clinicians and contribute to a significant burden on the health sector and diminishing quality of life among affected individuals (4).

**Figure 1:**
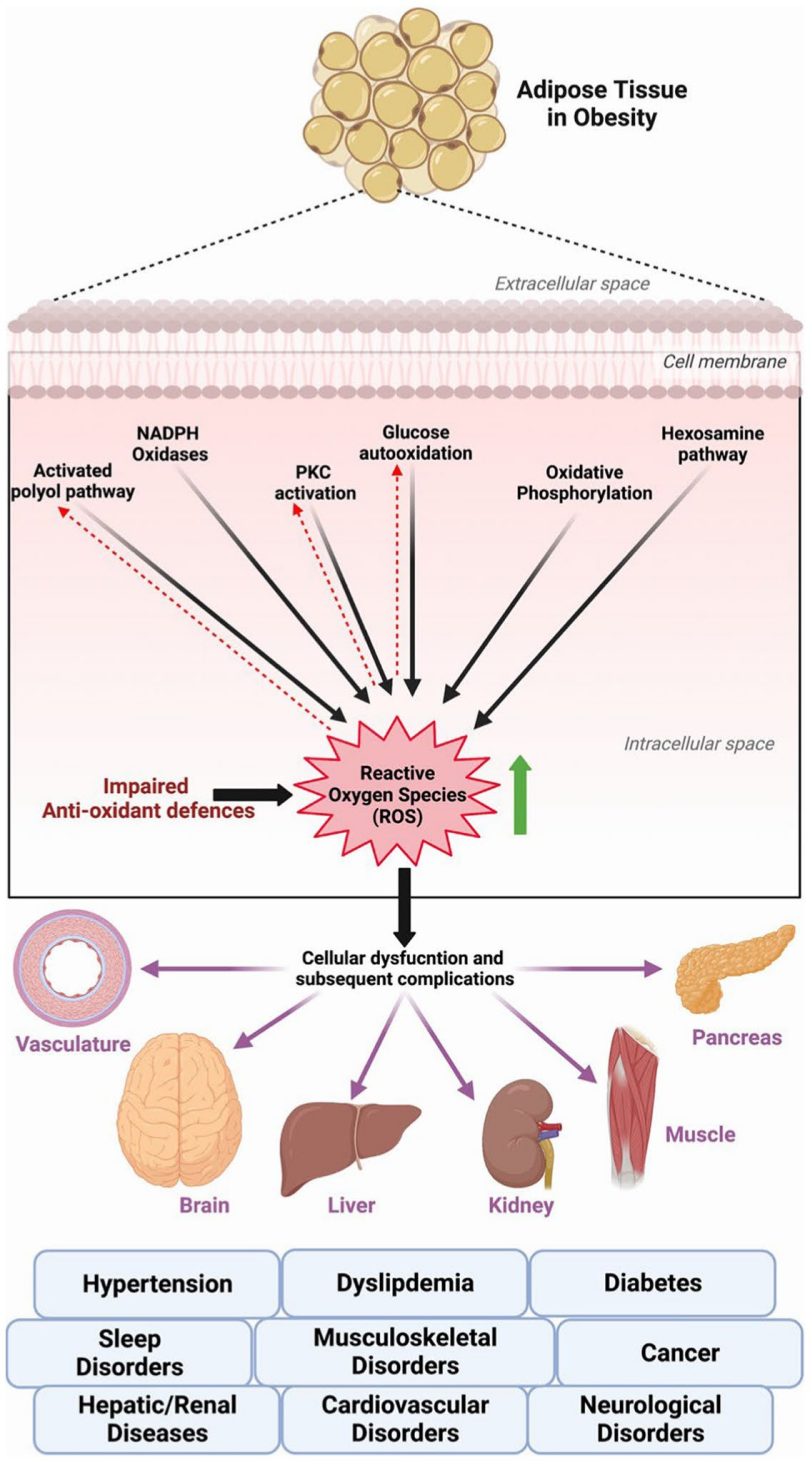
ROS generating pathways in obesity and related complications/diseases.

Obesity directly and adversely affects the functioning of several organ systems and increases the risk of developing diabetes, dyslipidemia, hypertension, cardiovascular diseases, musculoskeletal and neurological disorders, mood disorders, reproductive disorders, sleep disorders, hepatic and renal diseases, and several different cancers; ultimately all-cause death (3, 5) (Figure 1). Several obesity-related aberrant cellular, hormonal, and molecular changes can explain its relation to the increased risk of these comorbid conditions. However, growing scientific evidence links the excessive generation of reactive oxygen species (ROS) and subsequent oxidative stress and pro-inflammatory status in obese individuals as contributing factors to several illnesses and associated complications (6). ROS-generating mechanisms such as superoxide generation from NADPH oxidases, oxidative phosphorylation, glyceraldehyde auto-oxidation, protein kinase C activation, and polyol and hexosamine pathways contribute to the obesity-related cellular and systemic oxidative stress (6) (Figure 1). Hormonal imbalances (hyperleptinemia) and imbalances in the body’s antioxidant systems further aggravate the consequences of oxidative stress in obesity (6).

The major endogenous antioxidant mechanisms of human cells that scavenge the free radicals within the body are the catalases (Cat), superoxide dismutases (SOD), glutathione peroxidases (GPx), and the thioredoxin and glutardoxin antioxidant systems (7). In addition, several minerals (such as Se, Mn, Cu, Zn), vitamins (A, C, and E), and some other biomolecules (glutathione, bilirubin, uric acid) play crucial roles as antioxidants in the body (8). In healthy individuals, well-balanced antioxidant agents play an essential role in regulating the levels of free radicals, thus minimizing the detrimental effects of oxidative stress (8, 9). In obese individuals, however, the excessive production of free radicals coupled with an unresponsive antioxidant mechanism leads to a significant oxidative stress, a risk factor for obesity-related comorbidities and complications in the affected individuals (6).

In this regard, several synthetic pharmacological agents and naturally occurring compounds/phytochemicals have been widely studied for their antioxidant effect in treating obesity-related free radical generation, subsequent oxidative stress, and associated complications (10, 11). Among the naturally occurring compounds, flavonoids are one of the most widely studied compounds for their multifaceted beneficial effects, when used to treat obesity, diabetes, hypertension, CVD, and neoplasia (10, 11).

In the current manuscript, we delve into the oxidative stress-related adverse events that occur in obese individuals at the molecular level. We also explain how these aberrant molecular mechanisms relate to obesity-related comorbidities and complications among affected patients. Current anti-obesity treatment strategies, and the effect of flavonoid-based therapeutic interventions on digestion and absorption, macronutrient metabolism, inflammation and oxidative stress and gut microbiota has been evaluated. The therapeutic effects of different flavonoid compounds in reversing obesity and its related complications on a long-term basis are also reviewed.

## 2. Current obesity pharmacotherapy treatment strategies

Obesity is an increasing threat to human health worldwide. Diabetes, cardiovascular and locomotory diseases are the main comorbidities associated with obesity. It also significantly impacts the individual’s social and psychological status (12). Obesity is more considered as a chronic and degenerative disease rather than a risk factor (13). This has promoted raising funds for anti-obesity basic and clinical research and the establishment of health management programs (13).

Many factors govern obesity as a chronic, complex, and heterogeneous disease, such as genetic and environmental conditions; thus, approaches to treat obesity must be integrated and comprehensive (14). Anti-obesity treatment is recommended for individuals with body mass index (BMI) higher than 30 or higher than 25-27 with comorbidities (15, 16). The main challenge in obesity is to maintain long-term weight loss. This can be achieved through lifestyle modifications (diet, exercise, and behavioral therapy) and a pharmacological approach (14). A good anti-obesity drug should correct excess weight while reducing the risk of cardiovascular diseases, other co-morbidities, and the emergence of tachyphylaxis (17). Currently, anti-obesity treatment is based on various strategies. Drugs such as orlistat, naltrexone, phentermine/topiramate, and liraglutide, approved by the European agency, can promote long-term weight loss by decreasing fat absorption or suppressing appetite (17). Most anti-obesity drugs lead to a weight loss of about 3- 7%. Although paltry compared to the efficacy of bariatric surgery, such weight loss helps to improve the severity of comorbid diseases and is considered clinically meaningful (18).

Several sympathomimetics acting on the central nervous system, such as phentermine, cathine, and diethylpropion, were recommended only for short- term use in anti-obesity due to addictive potential or the emergence of tachyphylaxis (19). Phentermine, however, did not display adverse cardiovascular disorders and remains a commonly prescribed long- term drug (19).

Inhibition of digestive enzymes involved in the hydrolysis of lipids (lipases) and carbohydrates (amylases and alpha glucosidases) is another strategy to treat obesity (20). Orlistat (120 mg; approved since 1999) is an inactivator of pancreatic lipase (21). FDA-approved acarbose derived from Actinoplanes species as a pharmaceutical α-glucosidase inhibitor (22). Other prevention and treatment options could be based on strategies to dampen or inhibit intestinal nutrient absorption (14).

GLP1 (Glucagon-like peptide 1 receptor) and GIP (glucose- dependent insulinotropic polypeptide) are incretins, a group of metabolic hormones released after food intake in the blood to decrease glucose levels through stimulating insulin secretion and inactivating glucagon action (23). Although initially targeting diabetes, incretins were redirected as anti-obesity agents (24). GLP1R agonists like liraglutide (3 mg) and semaglutide are advanced therapeutic candidates for managing obesity. Semaglutide was approved by the FDA in 2021 for chronic weight management in adults, in addition to a reduced-calorie diet and increased physical activity (25). Liraglutide (3 mg) was approved by the FDA in 2014 for treating adult obesity and in 2020 for obesity in adolescents (12–17 years) and can achieve up to 14.9% weight loss (26). GLP1R agonists can also improve glycemia by enhancing insulin secretion and slowing glucose entry to general circulation upon inhibiting gastric emptying (27). Other studies showed that using poly-agonists simultaneously targeting GLP1, GIP (glucose-dependent insulinotropic polypeptide) and/or glucagon receptors gave promising results in mice (28). Many poly-agonist drugs are in clinical development (14). Leptin, ghrelin, mitochondrial uncouplers, and growth differentiation factor 15 (GDF15) are emerging anti-obesity agents (14). Leptin (discovered in 1994) regulates energy balance through peripheral hormone signals to the brain (29). Although inefficient when used alone in anti-obesity therapy, a combination of leptin and pramlintide induced more significant body weight loss than treatment with either drug (30). Plant-derived biomolecules such as celastrol (31) and withaferin A (32) decreased body weight by enhancing leptin sensitivity. Ghrelin is a gastric peptide hormone that stimulates food intake by acting on the hypothalamic feeding centers (33). Impairing ghrelin signaling and interaction with its receptor, the growth hormone secretagogue receptor (GHSR), might be a promising strategy to reduce hunger sensation and treat obesity. Mitochondrial uncouplers, such as 2,4-dinitrophenol (DNP), render the metabolism and production of ATP less efficient (34). Although DNP was banned from therapeutic use due to its adverse effects, the mitochondrial uncoupling route remains promising for developing anti-obesity drugs. Similarly, inhibiting the activation of the glial cell-derived neurotrophic factors (GDNF) family receptor α-like (GFRAL) by the macrophage inhibitory cytokine 1 (MIC1 or GDF15) was shown to suppress the appetite and to be a promising anti-obesity (35).

## 3. Anti-obesity effect of flavonoids by acting on digestion, absorption, and micronutrient metabolism

Polyphenols, especially flavonoids in plant foods, have attracted increasing interest due to growing evidence of their beneficial effect on human health (36). Over 10,000 plant flavonoids identified, including anthocyanidins, flavonols, flavanones, flavones, and isoflavones (37), have been identified to benefit the body. Flavonols such as quercetin and kaempferol are the most widespread flavonoids in plants. Dietary plants are the primary sources of flavones like luteolin and apigenin (36). Meanwhile, soybean-related foods contain high amounts of isoflavones such as genistein and daidzin issued from β-glycoside forms (38). Gastrointestinal enzymes involved in nutrient digestion and absorption are potential therapeutic targets for anti-obesity therapy (39). Dietary flavonoids can reduce fat and carbohydrate intake by regulating their hydrolysis and absorption in the gastrointestinal tract (40).

### 3.1. Inhibition of digestive enzymes

Lipases, α-amylases, and α-glucosidases are the main digestive enzymes whose inhibition can be used as a target for anti-diabetes and anti-obesity treatments (41). Preventing the digestion of complex carbohydrates through the inactivation of α-amylases and α-glucosidases leads to decreasing postprandial hyperglycemia (42). *In vitro* studies highlighted the inhibitory effects of flavonoids against lipid and sugar hydrolyzing enzymes. Polyphenols, including flavonoids, have been shown as good inhibitors of amylases (43). The methanolic extract of Solenostemma argel displayed a high inhibitory capacity against pancreatic lipase, α-amylases, and α-glucosidase (69-97%) (44). The extract displayed a high content of flavonoids. Likewise, Phenolic compounds (mainly flavonoids) extracted from edible cassava leaves were highly potent inhibitors against α-amylase, α-glucosidase, and lipase (45). Orientin, isoorientin, vitexin, and isovitexin, flavonoids extracted from *Phyllostachys edulis* leaf, displayed an *in vitro* α-amylase inhibitory activity. Molecular docking showed that the four flavones could bind to the α-amylase active site, and that binding involved the 3′-hydroxyl group and was influenced by the glucoside linking position (46). Similarly, Flavonoids apigenin, luteolin, kaempferol, naringenin, quercetin, myricetin, chrysin, and baicalein were described to display both α-amylase and α-glucosidase inhibitory capacity (47, 48).

**Figure 2:**
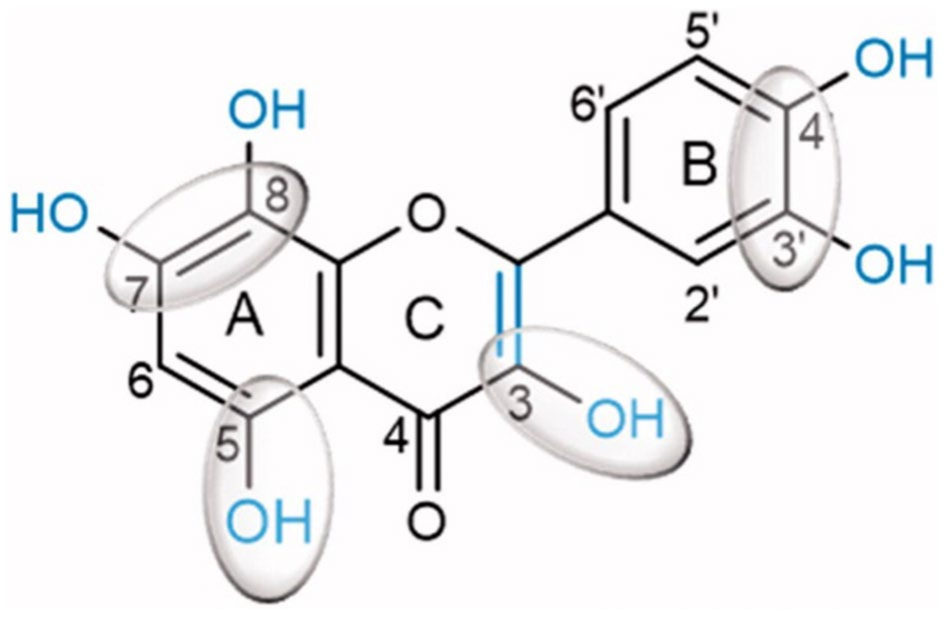
Quercetin chemical structure showing chemical determinants for flavonoid binding to glucosidase (51).

Inhibition of α-glucosidase, the most critical enzyme in carbohydrate digestion, aims to prevent excess glucose absorption in the small intestine. This helps to control blood glucose and prevent diabetes and obesity (49). Human α-amylases from both salivary and pancreatic origins were widely researched for clinical chemistry as targets for drug design to treat obesity and diabetes. Kaempferol, quercetagetin and galangin were better inactivators of α-glucosidases (sucrase, maltase and isomaltase) than flavan-3-ol(−)-epigallocatechin-3-gallate (EGCG) and quercetin (50). A structure–activity-based study on substituted flavonoids revealed that hydroxylations at positions 5- and 7- or 8- of the A-ring, positions 3’, 4’ of the B-ring and position 3 and C2 = C3 double bond in the C-ring, are critical for the inhibitory activity of flavonoids (Figure 2). This justified the high inhibitory potential of quercetin (51).

**Figure 3:**
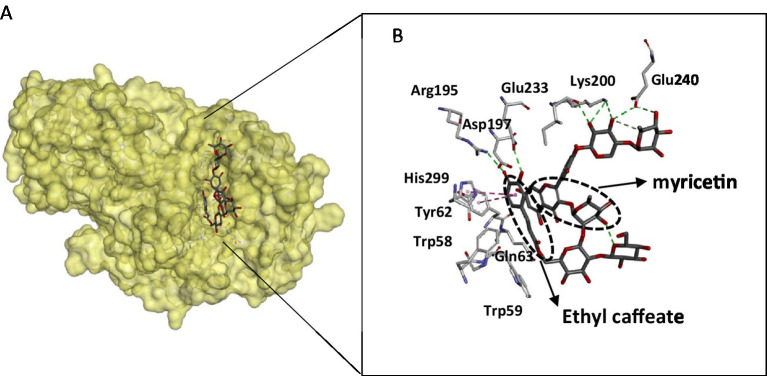
Structure of the pancreatic α-amylase complex with myricetin and ethyl caffeate (pdb code 4 W93). **(A)** The phenolic compounds within the catalytic pocket of the enzyme. **(B)** Interacting residues with phenolic compounds are displayed as sticks, and hydrogen bonding is shown as dotted lines. The figure was constructed using the BIOVIA Discovery Studio software (BIOVIA, Dassault Systèmes, [BIOVIA Discovery Studio], [v17.2.0.16349], San Diego: Dassault Systèmes, [2017]).

The crystal structure of the α- amylase in complex with montbretin (acylated flavonol glycoside containing myricetin and caffeate) revealed an internal π-stacking interaction of phenyl rings of myricetin and caffeic acid and orientation of their ring hydroxyls towards an optimal hydrogen bonding network with catalytic residues Asp197 and Glu233 (52). This binding mode mimics that of glycosides and adequately explains the inhibitory power of flavonols towards amylases (Figure 3). The flavonoid binds to the active site and interacts with catalytic residues, despite its planar structure, while ethyl caffeate acts through disordering loops nearby the active site cleft. Docking experiments of flavonoids: hesperetin, luteolin, quercetin, catechin, and rutin with α-amylase revealed interactions with key active site residues such as Trp 58, Trp 59, Tyr 62, Gln 63 that are involved in stacking interactions with the glycosyl substrate, in addition to catalytic Asp197, explain the inhibitory activity of these compounds (43). Interactions between flavonoids and α-amylase active site amino acid residues are mainly hydrophobic. The amylase crystal structure in complex with flavonols confirms that these aromatic residues are located close to the flavonoid binding site (Figure 3). This binding information is valuable for therapeutic developments targeting amylase.

**Figure 4:**
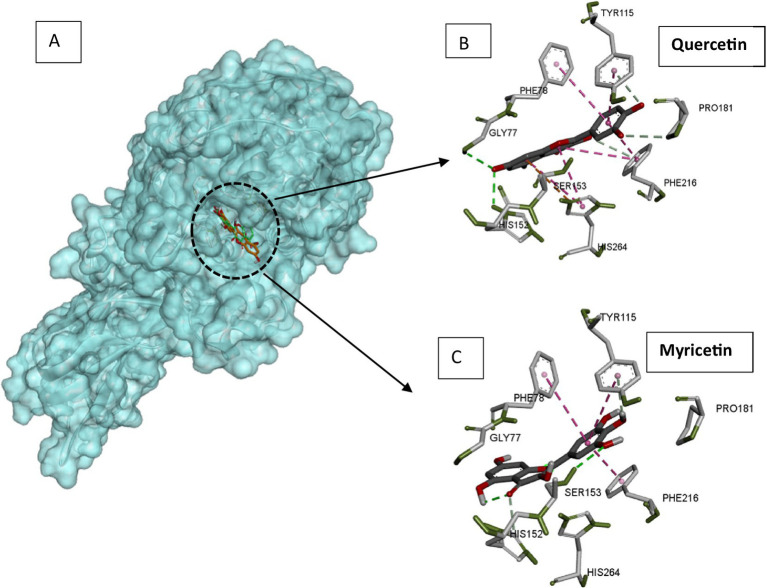
Docking models of pancreatic lipase with quercetin and myricetin. **(A)** The quercetin (green) and myricetin (orange) are superimposed and presented as sticks. **(B)** Interacting residues with quercetin in the lipase active site. **(C)** Interacting residues with myricetin in the lipase active site. Pancreatic lipase, quercetin, and myricetin structures were extracted from pdb structures with codes 1ETH, 6TTC, and 7VUM, respectively. Docking was carried out using autodock Vina software with a radius of 1.00, the center coordinates x: 64, y = 38.953 and z = 133.121, and a box size of 40. The figure was constructed using the BIOVIA Discovery Studio software (BIOVIA, Dassault Systèmes, [BIOVIA Discovery Studio], [v17.2.0.16349], San Diego: Dassault Systèmes, [2017]).

Pancreatic lipase is the main lipolytic enzyme target for anti-obesity treatments. Its inhibition can diminish fat levels in the blood, since this enzyme is responsible for the hydrolysis of approximately 70% of dietary fat (53). The major flavonoids in a citrus peel extract (hesperidin, naringin, neohesperidin, narirutin, and Eriocitrin) strongly inhibited pancreatic lipase *in vitro* (22). Catechin and rutin inhibit pancreatic lipase activity assayed on 4-nitrophenyl octanoate used as substrate (54). Likewise, quercetin was demonstrated to inactivate pancreatic lipase efficiently (55). Lipase mechanism inactivation by flavonoids was investigated through various structure–function relation studies. Flavones containing the C-glycosyl group at the C6 position with two sugar moieties were reported to display the highest pancreatic lipase inhibitory power. The presence of the C2 = C3 bond and the C=O group in C ring and the hydroxylation of A and B rings are flavone structural features that enhance the inhibitory power towards lipases. Quercetin displayed a strong inhibitory activity towards the pancreatic lipase, while capsaicin was a poor inhibitor (56). The docking experiment showed quercetin to bind within the active site and capsaicin far from the catalytic cavity (56). The model of the complexes between pancreatic lipase and quercetin or myricetin was generated by docking using auto dock vina software (57). The generated complexes displayed a good superimposition of flavonoid positions within the lipase active site (Figure 4A). Through their phenyl rings, both flavonoids were establishing stacking interactions with hydrophobic aromatic active site residues (Phe78, Pro181, Tyr115, and Phe216), but also with catalytic His264 (Figures 4B,C). Ligands were located close to the catalytic serine (Ser153), to which myricetin is hydrogen bonded (Figure 4C). All these interaction features of flavonoids inside the lipase active site explain the inhibitory power of quercetin and myricetin exerted on the enzyme, lowering fat processing, and reducing fat uptake.

### 3.2. Inhibition of nutrient absorption

Damping nutrient absorption through the intestinal membrane is an emerging strategy for developing anti-obesity agents. Clinical research used similar strategies to diminish the absorption of cholesterol, fat, and carbohydrates (58). GLUT proteins are members of the solute carrier (SLC) family responsible for transporting monosaccharides and polyols across cell membranes. Intestinal glucose absorption across the apical membrane involves sodium-glucose cotransporter 1 (SGLT1) and GLUT2, is involved in glucose transport to the blood through the basolateral membrane (59).

Flavonoids such as tiliroside, myricetin, phloretin, EGCG, and apigenin were reported to inactivate GLUT2 (60, 61). Quercetin inhibits glucose and fructose transport by GLUT2, binding to a non-sugar binding site. Meanwhile, epicatechin-gallate (ECG) and epigallocatechin-gallate (EGCG) impair sugar uptake by GLUT5 (62). Likewise, apigenin and EGCG inhibited glucose and fructose transport by GLUT2 and LUT5 (63). Meanwhile, apigenin was more effective than EGCG or quercetin in inactivating glucose and fructose transport by GLUT7.

### 3.3. Effects of obesity on macronutrient metabolism and protective role of flavonoids

The effect of obesity on macronutrient metabolism is closely related to the ongoing insulin resistance in obese individuals, which is characterized by resistance to the effect of insulin on glucose uptake, utilization, and storage (64).

#### 3.3.1. Carbohydrate metabolism

In normal individuals, insulin, produced by the pancreatic β-cells, maintains normal blood glucose levels by the activation of hepatic glycogen synthesis, inhibiting hepatic gluconeogenesis and increasing cellular glucose transporter 4 (GLUT4) mediated glucose uptake by the muscle and adipose tissue (64, 65). The translocation of GLUTs to the plasma membrane in response to insulin is mediated by insulin binding to its specific cell surface receptors, subsequent phosphorylation of insulin receptor substrates 1 and 2 (IRS-1 and 2), which in turn activates the PI3K/Akt and MAPK pathways (66).

The skeletal muscle was once considered the central tissue for insulin-mediated glucose uptake and maintaining glucose homeostasis (64). However, studies have revealed that the adipocytes play a major role in insulin-stimulated GLUT 4-mediated glucose uptake into the fat tissue (67). Selectively knocking down GLUT4 in adipocytes resulted in insulin resistance similar to that observed when knocking down the glucose transporter in skeletal muscle cells (68). On the other hand, overexpression of GLUT4 in fat tissue increased glucose tolerance and improved overall sensitivity to insulin (67).

In obese individuals, GLUT4 was significantly downregulated in adipocytes compared to non-obese individuals. It was linked to impaired glucose uptake in the fat tissue, while levels of GLUT4 in the skeletal muscle remain unchanged (69). Impairment of GLUT4 translocation, docking, and fusion with vesicles contributed to defective cross-membrane glucose transport. Interestingly, oxidative stress repressed GLUT4 mRNA expression and protein levels in adipocytes while using antioxidants such as lipoic acid protected against oxidative stress-mediated in GLUT4 (70). Insulin insensitivity and prolonged insulin exposure and stimulation also caused an oxidative stress-mediated downregulation of GLUT4 of 3 T3-L1 adipocytes (71).

#### 3.3.2. Lipid metabolism

Classical dyslipidemia, observed in approximately 60-70% of obese individuals, is characterized by elevated serum levels of triglycerides, VLDL, apolipoprotein-B (Apo B), and non-HDL-cholesterol (HDL-C) and low serum levels of HDL-C levels and Apo A1 (72, 73). While LDL-C levels remain within the normal range, the increased formation of pro-atherogenic small dense LDL-C particles is observed in obese individuals compared to normal individuals (72). Elevated postprandial pro-atherogenic plasma triglyceride (TG) levels (hypertriglyceridemia), owing to the increase in TG production and decrease in TG lipolysis, were reported in 60-70% of obese individuals (72).

The various abnormalities that lead to dyslipidemia seen in patients with obesity are triggered and driven by the increased availability of free fatty acids (FFA) to the liver due to increased adiposity, insulin resistance, and inflammation (74, 75). Increased basal levels of lipolysis and circulating free fatty acids are hallmarks of altered lipid metabolism in obese individuals (64). The enlarged adipocytes in obese individuals are known to secrete the serum amyloid A (SAA) protein causing insulin resistance which, via an autocrine feedback loop, upregulates SAA-dependent lipolysis in the adipose tissue either directly via the ERK signaling pathway or indirectly via the upregulation of pro-inflammatory cytokines such as IL-6 and TNFα (64, 76, 77). Obesity-related increase in FFA flux in the adipose tissue due to increased TG breakdown (owing to insulin resistance), increased de-novo synthesis of FFA in the liver, and uptake of triglyceride-rich lipoproteins by the liver are the major sources of excess FFA in obese individuals (74, 75).

Obesity-related excessive ROS production and oxidative stress increase the accumulation of lipid peroxidation products in obese individuals and thus contribute to the severity of the metabolic syndrome (78–80).

#### 3.3.3. Protein metabolism

It is well-known that obesity-related insulin resistance and oxidative stress alter normal carbohydrate and lipid metabolism. However, much less appreciated is the fact that obesity and insulin resistance can change protein metabolism in obese individuals (64).

Although protein metabolism at the whole-body level is less sensitive to insulin action, the obesity-related effect related to insulin resistance and its impact on the locomotor, weight-bearing, and skeletal muscle protein cannot be ignored (81). Increased proteolysis and increased rate of basal leucine turnover linked to the impairment of insulin’s anti-proteolytic effect were reported in moderately obese individuals (64). Studies have reported aberrant protein synthesis rates in skeletal muscle in the post-absorptive state in obese subjects (82). Impairment of the activation of skeletal muscle protein synthesis in response to nutrient intake was reported in a C57BL/6 J mouse model of diet-induced obesity (83).

Mechanistically, insulin secretion-related increase in the plasma amino-acid concentrations stimulates overall muscle protein synthesis via the activation of the mTOR pathway and vice versa (82, 84). Under physiological conditions, insulin triggers the Akt/mTOR pathway and increases muscle protein synthesis (82). On the other hand, insulin resistance in obesity can be correlated to decreased activation of the Akt/mTOR pathway and hence reduced muscle protein synthesis (85–87). In addition, impaired vasodilation in obese insulin-resistant individuals and blood flow restriction can negatively impact amino acid delivery into the skeletal muscle and thus reduce protein synthesis (88–90). Obesity-related insulin resistance, inflammation, and oxidative stress impair anabolic stimulant-responsive protein remodeling in skeletal muscles, thus contributing to poor muscular health and fitness in obese subjects (81, 91).

#### 3.3.4. Role of flavonoids in the regulation of macronutrient metabolism

Several dietary flavonoids improve insulin sensitivity, inhibit obesity-related oxidative stress, improve the redox balance in affected individuals and thus improve macronutrient metabolism (92). Some essential dietary flavonoids that possess anti-obesity and/or insulin-sensitizing effects that impact oxidative stress and macronutrient metabolism (data based on *in vitro* and/or *in vivo* studies) are provided in Table 1. Table 2 shows clinical trials that studied the effect of flavonoids/dietary flavonoids on insulin resistance in type 2 diabetes and obesity.

**Table 1:** Important dietary flavonoids with anti-obesity and/or insulin-sensitizing effects that impact oxidative stress and macronutrient metabolism.

Flavonoids/*In vitro*/*In vivo* and dosage/concentration	Dietary Source	Anti-obesity and/or insulin sensitizing effect	Key molecular changes/mechanisms	References
**Apigenin**				
*In vitro*, HIT-T15 pancreatic β-cells, 10 μM apigenin *In vivo*, STZ induced diabetic male Wistar rats, 4.0 mg apigenin /kg BW	Parsley, chamomile, celery, vine, spinach, artichoke and oregano	↑Glucose uptake, ↓Lipid accumulation, ↓Hyperglycemia, ↑Insulin secretion and sensitivity, ↓Oxidative stress	↑GLUT4, ↑AMPK, ↑ACC, ↓G6Pase, ↓MCP1, ↓NF-κB, ↓IL6, ↓TNFα	(93, 94, 95)
**Eligallocatechin Gallate (EGCG)**				
*In vivo*, High fat diet fed C57BL/6J mice, 3.2 g EGCG/kg of diet *In vivo*, High fat diet fed Sprague–Dawley rats, 3.2 g EGCG /kg of diet *In vivo*, male Wistar rats, 5.0 mg EGCG/kg BW	Green tea, other tea varieties (white, oolong, black), berries, pears, peaches, apples, avocados, pecans, pistachios, and hazelnuts	↑Insulin secretion and sensitivity, ↑Protection of insulin-producing pancreatic β-cells, ↓Glycemia, ↓Insulinemia, ↓HOMA-IR, ↓BW, ↓FFA	↑FOXO1, ↑PDX1, ↑IRS2, ↑AKT, ↓IRS1, ↑PI3K, ↑GLUT4	(66, 93, 96, 97, 98)
**Epicatechin Gallate (EGC)**				
*In vitro*, HepG2 cells, 50 μM EGC	Tea, grapes, and seeds of leguminous plants	↓Hepatic lipid accumulation	↓Fatty acid synthase, ↓ACC1	(93, 99)
**Genistein**				
*In vitro*, HAEC cells, 0.01-10 μM genistein *In vitro*, 3T3-L1 preadipocyte cells, 100 μM genistein *In vivo*, db/db diabetic mice (B6.Cg-m^+/+^Lepr^db^), 0.1% genistein in diet *In vivo*, STZ induced diabetic male C57BL/6J mice, 0.025 g% genistein in diet *In vivo*, STZ induced diabetic male Wistar rats, 300 mg genistein /kg BW/day *In vivo*, Albino Swiss mice, 1 mg genistein /kg BW *In vivo*, High fat high fructose diet fed Albino Swiss mice, 1.0 mg genistein/kg BW/day	Soy-based foods	↓Plasma triglyceride, ↑Insulin producing pancreatic β-cells, ↓Hyperglycemia, ↓HbA1c, ↓Adipocyte differentiation, ↓HOMA-IR, ↓Hepatic lipid accumulation; ↑Insulin signaling, ↓Glycemia, ↓Insulinemia, ↓BW, ↓ FFA, ↓ TG, ↓ TC	↑AMPK, ↑ACC, ↑GLUT4, ↑cAMP signaling, ↑PKA activation, ↓TNFα, ↓TGFβ, ↓NF-κB, ↑AKT	(66, 93, 100, 101, 102, 103)
**Hesperidin**				
*In vivo*, STZ induced diabetic male Wistar rats, 10.0 g hesperidin/kg of diet *In vivo*, C57BL/KsJ db/db mice, 0.2 g hesperidin/kg of diet	Orange, mandarin, lemon, lime, grapefruit	↓Hyperglycemia, ↓Oxidative stress, ↓Hyperlipidemia,	↑GLUT4, ↓HMGCoA, ↓ACAT	(93, 104, 105)
**Kaempferol**				
*In vitro*, INS-1E pancreatic β-cells, 0.1–10 μM kaempferol *In vivo*, High fat diet fed male C57BL/6J mice, 0.15 g% kaempferol in diet	Cruciferous vegetables (spinach, kale), herbs (dill), grapefruit, tea	↓Hyperglycemia, ↑Muscle glucose uptake, ↑β-cell survival, ↑Antioxidant defence, ↓Oxidative stress	↓Caspase 3, ↑GLUT4, ↑AMPK, ↓PPARγ, ↓SREBP-1c, ↓TNFα, ↓IL6	(93, 106, 107)
**Luteolin**				
*In vitro*, 3 T3-L1 preadipocyte cells, 20.0 μM luteolin *In vitro*, Isolated mouse epididymal adipocyte cells C57BL/6J mice, 20.0 μM luteolin *In vitro*, Isolated pancreatic islet β-cells from C57BL/6J mice, 10.0 μM luteolin *In vitro*, Min6 insulin secreting cells, 10.0 μM luteolin	Celery, parsley, broccoli, onion leaves, carrots, pepper, cabbages, apple skin, chrysanthemum flowers	↑Insulin secretion and sensitivity	↑GLUT4, ↑Leptin, ↓PPARγ, ↓NF-κB, ↓CREB-B	(93, 108, 109)
**Naringenin**				
*In vivo*, High cholesterol diet fed male Sprague–Dawley rats, 0.1% naringenin w/w in diet *In vivo*, STZ induced diabetic male Wistar rats, 10.0/20.0/50.0 mg naringenin/kg BW *in vitro*, 3 T3-L1 preadipocyte cells, 25-100 μM naringenin	Grapefruit, sour orange, tart cherries, tomatoes, Greek oregano	↓Hyperlipidemia, ↓Fatty liver, ↓Hyperglycemia	↓HMGCoA, ↓ACAT, ↓PI3K, ↓AKT, ↑SOD	(66, 93, 100, 101, 102, 103)
**Quercetin**				
*In vitro*, 3 T3-L1 fibroblast cells, 12.5 or 25 μM quercetin in combination with 25 μM resveratrol *in vivo*, High-fat high-sucrose diet fed male Sprague–Dawley rats, 0.02/0.07/0.5% quercetin w/w in diet *In vivo*, Alloxan induced diabetic Swiss albino mice, 20 mg quercetin/kg BW *In vitro*, FL83B cells, 6 μg/ml quercetin *In vivo*, Fructose-rich diet fed C57BL/6J mice, 3 mg quercetin/kg BW	Citrus fruits, berries, apples, onions, parsley, sage, tea, red wine, olive oil, grapes	↑Pre-adipocyte apoptosis, ↑Adipocyte and muscle glucose uptake, ↓Pre-adipocyte fat-accumulation, ↓Hyperglycemia, ↑Insulin secretion and sensitivity	↑AMPK, ↑Caspase 3 and 9, ↑GLUT4, ↓PARP, ↓PPARγ, ↓NF-κB, ↓MDA, ↑SOD and catalase	(93, 110, 111, 112)
**Resveratrol**				
*In vitro*, HepG2 cells, 10 and 50 μM resveratrol *In vitro*, 3 T3-L1 preadipocyte cells, 50 μM resveratrol *In vitro*, Rat adipocytes isolated from male Wistar rats, 125 and 250 μM resveratrol	Red wine, grape juice, grapes, peanuts, cocoa, berries	↑Glucose uptake, ↓Lipid accumulation, ↑Adipocyte lipolysis	↑GLUT4, ↓PARP, ↑cAMP	(93, 113, 114, 115)
**Rutin**				
*In vitro*, FL83B cells, 23 μg/ml rutin *In vivo*, Fructose-rich diet fed C57BL/6J mice, 11.5 mg rutin/kg BW *In vivo*, High-fat diet fed male Wistar rats, 50 or 100 mg rutin/kg BW *In vitro*, 3 T3-L1 preadipocyte cells, 0.25/0.5/1.0 mg/ml *In vivo*, High-fat diet fed C56BL/6J mice, 25 or 50 mg rutin/kg BW	Buckwheat, asparagus, unpeeled apples, figs, black tea, green tea, and elderflower tea.	↓Hyperlipidemia, ↓Fatty liver, ↑Muscle glucose uptake	↑PI3K, ↑MAPK, ↓PPAR and C/EBP, ↓IL6, ↓TNFα	(93, 110, 116)

Data based on *in vitro* and/or *in vivo* studies. Table adapted from Hossain et al. (93) and Martin et al. (117). The upward arrow (↑) indicates an increase and the downward arrow (↓) indicates a decrease. ACAT: Acetyl CoA acetyl transferase; ACC: Acetyl CoA carboxylase; SREBP-1c: Sterol regulatory element binding protein 1c; AKT: Protein kinase B; AMPK: Adenosine monophosphate activated protein kinase; BW: Body weight; C/EBP: CCAAT enhancer binding protein; cAMP: cyclic adenosine monophosphate; CREB: cAMP responsive element binding protein; FFA: Free fatty acids; FOXO1: Forkhead Box O1; G6Pase: Glucose 6 phosphatase; GLUT4: Glucose transporter 4; HbA1c: glycosylated hemoglobin; HMGCoA: 3-hydroxyl-3-methylglutaryl-CoA reductase; HOMA-IR: homeostasis model assessment of insulin resistance; IL6: Interleukin 6; IRS1/2: Insulin receptor substrate 1/2; MAPK: Mitogen activate protein kinase; MCP1: Monocyte chemoattractant protein 1; MDA: Malondialdehyde; NF-κB: Nuclear factor kappa B; PARP: Poly-ADP ribose polymerase; PDX1: Pancreatic and duodenal homeobox 1; PI3K: Phosphatidylinositol-4, 5, bisphosphate 3-kinase; PPAR: Peroxisome proliferator activated receptor; PPARγ: Peroxisome proliferator activated receptor gamma; SOD: Superoxide dismutase; STZ: Streptozotocin; TC: Total cholesterol; TG: Triglyceride; TGFβ: Transforming growth factor beta; TNFα: Tumor necrosis factor alpha.

**Table 2:** Clinical trials on the effect of flavonoids/dietary flavonoids on insulin resistance and type 2 diabetes and obesity.

Treatment	Number of participants	Metabolic effect	Study type	References
850 mg flavanols and 100 mg isoflavones/day, 1 year	93 females, type 2 diabetic	=Glycemia, =HbA1c, ↓Insulinemia, ↓HOMA-IR, ↓LDL, =HDL	Randomized controlled double-blinded, parallel	(66, 118)
1,500 mg of green tea extract (856 mg of ECGC)/day, 16 weeks	35 males, 33 females, type 2 diabetic	=Glycemia, ↓Insulinemia, ↓HOMA-IR, ↑HDL, =LDL, =TG	Randomized controlled double-blinded, parallel	(66, 119)
83.6 mg of cocoa flavanols, acute	3 males, 9 females, hypertensive type 2 diabetic	=Glycemia, =Insulinemia, =HOMA-IR, =LDL, =HDL, =TG	Randomized controlled double-blinded, crossover	(66, 120)
Cocoa beverage (960 mg polyphenols), acute	4 males, 14 females, type 2 diabetic	=Glycemia, “Insulinemia, =HOMA-IR, ↑HDL, =LDL, =TG	Randomized controlled double-blinded, crossover	(66, 121)
83.6 mg of cocoa flavanols/day, 12 weeks	18 males, 17 females, hypertensive type 2 diabetic	=Glycemia, =HbA1c, =Insulinemia, =HOMA-IR, =LDL, =HDL, =TG	Randomized controlled double-blinded, parallel	(66, 122)
Dark chocolate (450 mg flavanols)/day, 8 weeks	7 males, 5 females, hypertensive type 2 diabetic	=Glycemia, =HbA1c, =Insulinemia, =HOMA-IR, ↑HDL, =LDL	Randomized controlled double-blinded, crossover	(66, 123)
135 mg silybin/day, 6 months	20 males, 22 females, type 2 diabetic	↓Glycemia, =Insulinemia, =HOMA-IR, =HDL, ↓TG	Randomized controlled double-blinded, parallel	(66, 124)
Grape seed extract (600 mg of flavonoids)/day, 4 weeks	16 males, 16 females, type 2 diabetic	=HOMA-IR, =HDL, =TG, ↓TC	Randomized controlled double-blinded, crossover	(66, 125)
600 g blackberries (1,500 mg flavonoids), acute (12 h)	27 males (BMI > 25 kg/m2, 53–63 years)	=Glycemia, =Insulinemia, ↓HOMA-IR, ↓HOMA-B, ↓Respiratory quotient, ↓TG	Randomized controlled, crossover	(66, 126)
Kosen-cha (1 L/day, 14,300 mg polyphenols), 12 weeks	4 males, 2 females, (BMI = 25–35 kg/m2, ≥45 years)	=Glycemia, ↓HOMA-IR, ↓BW, ↓BMI, ↓WC, ↓TG	Prospective	(66, 127)
Pecan-rich diet (15% of total calories), 12 weeks	21 males, 5 females, (BMI > 25 kg/m2, 38–58 years)	↓Glycemia, ↓Insulinemia, ↓HOMA-IR, ↓HOMA-B, VLDL, ↓LDL, ↓HDL, ↓TG	Randomized controlled blinded, crossover	(66, 128)
Pomegranate juice (500 ml, 1,685 mg polyphenols/L), 4 weeks	12 males, 16 females, (BMI = 25–35 kg/m2, 40–65 years)	↓Insulinemia, ↓HOMA-IR = BW, =BMI	Randomized controlled, crossover	(66, 129)
Dark chocolate (20 g, 500 mg polyphenols), 4 weeks	42 females (25 overweight [BMI 25 kg/m2] + 21 controls, [BMI = 18–24.9 kg/m2])	=Insulinemia, =HOMA-IR, ↓BW, =WC	Randomized controlled, single-blinded, crossover	(66, 130)
Orange juice (750 ml/day, 135 mg flavonoids/L), 8 weeks	46 females (13 overweight +8 obese, [BMI 25 kg/m2] + 21 controls, [BMI = 25 kg/m2])	=Glycemia, =Insulinemia, =HOMA-IR. ↓TC, ↓LDL, =HDL, =BMI, =body fat mass, =body mass, =WC	Randomized controlled double-blinded, parallel	(66, 131)
Green decaffeinated tea (960 ml/day, 235.64 mg catechin and 128.84 mg EGCG), 6 months	54 females, breast cancer survivors (BMI = 25–40 kg/m2, 18–80 years)	=Glycemia, =Insulinemia, =HOMA-IR, ↓LDL, ↑HDL, =TG, ↓BW, ↓BMI, ↓fat mass, ↓lean mass, ↓WC, ↓hip circumference	Randomized controlled, double-blinded, parallel	(66, 132)

Table adapted from Martin et al. (117). The upward arrow (↑) indicates an increase, the downward arrow (↓) indicates a decrease and the equal sign (=) indicates unchanged parameters. BMI: body mass index; BW: body weight; HbA1c: glycosylated hemoglobin; HDL: high-density lipoprotein; HOMA-B: homeostasis model assessment of β-cell function; HOMA-IR: homeostasis model assessment of insulin resistance; LDL: low-density lipoprotein; TC: total cholesterol; TG: triglycerides; VLDL: very low-density lipoprotein; WC: waist circumference.

## 4. Anti-obesity effects of flavonoids mediated by anti-oxidative and anti-inflammatory properties

Research and clinical studies have provided evidence for the health benefits of flavonoids in treating and preventing diabetes due to their strong antioxidant and anti-inflammatory properties (133). A study done in Korea on 23,118 adult individuals provided evidence that flavonoid intake was associated with a lower prevalence of abdominal obesity and percent body fat in women (134). A separate trial conducted by Munguia et al. (135), provided evidence that the oral administration of cocoa supplements enriched in flavonoids reduced biomarkers of oxidation and inflammation in middle-aged and older patients.

### 4.1. Oxidation

Oxidative stress is a feature of many pathologies, including obesity. Excessive lipid accumulation leads to increased markers of oxidative stress and metabolic changes in adipose tissue (136). Reactive oxygen species (ROS) can lead to mitochondrial dysfunction in adipose tissue, resulting in increased triacylglycerol accumulation and a reduction in energy expenditure; the accompanying increase in pre-adipocyte differentiation into mature adipocytes means that ROS can lead to obesity (137, 138). Moreover, in obese individuals, there is an 80% prevalence of non-alcoholic fatty liver disease (NALFD). In this condition, the liver is a target tissue for ROS because the increased influx of free fatty acids into the liver leads to oxidative phosphorylation and ATP generation, contributing to the formation of ROS (139, 140).

Flavonoids can act as natural antioxidants, modulating oxidative stress, neutralizing reactive oxygen and nitrogen species, and thus helping prevent obesity (141). The phenolic flavonoid compounds have a well-detailed antioxidant activity and can act to scavenge ROS and RON, either directly (142) or indirectly through activating endogenous antioxidant defense pathways, such as the Keap1-Nrf2-ARE pathway (143).

There is also evidence to suggest that ingesting flavonoids can elevate PPAR- α gene expression, which regulates hepatic lipid metabolism by enhancing beta and omega oxidation (144). PPAR- α can also promote the oxidation of fatty acids by enhancing the transcription of the carnitine palmitoyl transferase 1 (CPT-1) gene, the main regulatory enzyme in the oxidation of long-chain fatty acids (LCFA) (145). The upregulation of the genes PPAR-α, and CPT-1 can also increase fatty acid oxidation in the liver and activation of lipogenic enzymes, such as CPT, that help accelerate lipid influx into mitochondria for oxidation (146).

A biochanin (Table 3), isoflavone helps mitigate HFD-induced hepatic steatosis and IR by decreasing lipogenesis and fatty acid synthesis and increasing the expression of proteins involved in the oxidation of fatty acids in the livers of obese mice (147). Another flavonoid cyanidin-3-glucoside (C3G) (Table 3) improves glucose homeostasis and insulin sensitivity by reducing hepatic fat accumulation through modulating genes involved in the synthesis (PGC1α) and oxidation (Cpt-1α) of fatty acids (149).

**Table 3:** *In-vivo* effects of flavonoids on oxidation in various models of obese mice.

Flavonoids/mice used	Dietary source	Duration and dosage	Key molecular changes/mechanisms	References
Biochanin HFD/male C57BL/mice	Red clover, soybeans, certain fruits, legumes, and nuts.	12 weeks, 0.05 % (w/w)	↓ Lipogenesis and fatty acid synthesis ↑ Proteins involved in oxidation of fatty acids	(147)
Apigenin HFD/male C57TBL/6J mice	Parsley, chamomile, celery, vine-spinach, artichokes, and oregano.	3 weeks, 30 mg/kg	↓ MCP-1, TNF-α ↑Nqo1, Gclc, Gstm1, Gsta2, Gclm, Gsta4, SOD, CAT, GPH-Px, TBARS ↑ Keap1 and NRF2	(148)
Cyanidin-3-glycoside male C57BLKS/J-Leprdb/Leprdb mice	Black elderberries, rubus, and bilberries.	16 weeks, 1 g/ml	↓ IL-6, MCP-1, TNF-a in liver. ↑ TFAM, NRF1, NRF2 in brown and white adipose tissue.	(149)
Naringenin HFHCD/male mice	Grapefruits, sour orange, tart cherries, tomatoes, and Greek oregano.	12 weeks, 3% (w/w)	↓ CD68+ cells, CD45+, CD115-, GR-1+ cells, CD35+, CD115+, CD45+, CD115+, Lin-, Sca-1+	(150)
Hesperidin NA	Orange, mandarin, lemon, lime, grapefruit	↓Hyperglycemia, ↓Oxidative stress, ↓Hyperlipidemia,	↑GLUT4, ↓HMGCoA, ↓ACAT	(93, 104, 105)
Silymarin HFD/male C57TBL/6J mice	Milk Thistle	30 days, 30 mg/kg	↓ p40-phox, p47-phox, p67-phox, NOX2, NOX4, NOXO1, NO, p47-phox, p67-phox, TRL4, iNOS, P65 nucleous. ↑ P65 cytoplasm and IkBa.	(151)
Myricetin HFD/male C57TBL/6J mice	Vegetables, fruits, nuts, berries, herbs, and tea, wine, fruit, and medicinal plants.	12 weeks, 0.12 % (w/w)	↑ Nuclear Nrf2, NQ01, HO-1, SOD2, CAT, GPx3, Prdx1, Prdx5 and TBARS in the liver. ↓ Cytosolic Nrf2 in the liver.	(152)
Morin HFD/male ICR mice	Figs, sweet chestnut, jack fruit, red wine, seaweed, tea, coffee, and cereal grains.	6 weeks, 50 mg/kg	↓ MCP-1 and MDA in serum. ↓ MDA, SOD and CAT in liver.	(153)
Morin HFD/male ICR mice	Figs, sweet chestnut, jack fruit, red wine, seaweed, tea, coffee, and cereal grains.	6 weeks, 100 mg/kg	↓ MCP1 and IL-6 in serum.	(153)
Quercetin WD/male C57TBL/6J mice	Apples, onions, parsley, sage, tea, and red wine.	20 weeks, 0.05 % (w/w)	↓ TNF-α in the plasma. ↓ TBARS in the liver. ↑ Gpx1 and CAT in the liver too.	(154)
Quercetin HFD/male C57BL/6J mice	Apples, onions, parsley, sage, tea, and red wine.	16 weeks, 0.05 % (w/w)	↓ IL-6, TRL-4, p65 nuclear, TNF-a, IL-6, NLRP3, caspase 1, GRP78, CHOP. ↑p65 cytosolic.	(155)
Quercetin HFD/male C57BL/6J mice	Apples, onions, parsley, sage, tea, and red wine.	6 weeks, 20 mg/kg	↓TNF-a, CDC8, MCP-1 in serum. ↓ p-JNK/JNK, TRL4, ATF-6, XPB-1 in white adipose tissue.	(156)
Troxerutin HFD/male C57TBL/6J mice	Tea, coffee, cereal grains and vegetables.	20 weeks, 150 mg/kg	↓ p65 nuclear, p-PERK, p-eIF2, pIRE1, TRAF2, NOD1, NOD2, 4-HNE, IL-1β, TNF-α, MCP-1 in the liver. ↑p65 cytosolic, GSH, SOD1, CAT in the liver.	(157)

The upward arrow (↑) indicates an increase and the downward arrow (↓) indicates a decrease in the expression or activity. CD; Cluster of Differentiation: GLUT; Glucose Transporters; HFD: High Fat Diet; HFHCD: High-Fat-High-Cholesterol-Diet; IL: Interleukins; MCP: Monocyte chemoattractant protein; SOD: Superoxide Dismutase; TBARS: Thiobarbituric reactive substances; TNF: Tumor Necrosis Factor; WD: high-fat, high-cholesterol and high sucrose western style diet; (w/w): weight for weight.

Another study evaluating the effect of treatment with the Flavonol Morin (Table 3) for 6 weeks in obese mice fed an HFD observed a reduction in oxidative stress indicated through lower serum and hepatic levels of MDA. An increase in liver expression of the antioxidant enzymes SOD and CAT was also seen. Morin’s antioxidant activity was derived from its improvement of glucose and lipid metabolism and attenuation of the inflammatory state induced by the HFD (153).

A flavanone, Silymarin (Table 3), showed oxidative stress reduction through the restoration of activities of antioxidant enzymes (SOD, CAT, and GPx) in the plasma and/or liver of animals fed with an HFD (151). The authors of this research suggested that the antioxidant activity of Silymarin was responsible for mitigating body weight gains, improving glucose intolerance and IR in the HFD mice (151).

Feng et al. (148) demonstrated that apigenin (Table 3) is effective in modulating the Keap1-Nrf2-ARE pathway; this modulation results in the regulation of genes involved in the synthesis and oxidation of FA, attenuating the accumulation of lipids in hepatocytes, and promoting an endogenous antioxidant response in the liver. In mice supplemented with apigenin, an increase in the activity of the antioxidant enzymes SOD, CAT, and GSH-Px was observed compared to the non-supplementary group (148).

A Naringenin (Table 3) supplementation showed to increase the expression of genes involved in the mobilization and oxidation of fatty acids, leading to reduced lipid accumulation in hepatocytes and attenuating hepatic steatosis- one of the contributors to the progression of atheromatous plaque (150). Quercetin and rutin (Table 3), quercetin’s aglycone form, were shown to increase the expression of antioxidant genes such as GPx and CAT, reduce levels of thiobarbituric acid reactive substances (TBARS) and increase gluthanione levels (154).

Some mice that were fed a high-fat diet (HFD) and were put on myricetin (Table 3) treatment for 12 weeks; a reversal of some of the metabolic changes caused by the diet was seen (152). A reduction in oxidative stress mediated by the Nrf2 pathway was observed, accompanied by an increase in the activity of antioxidant enzymes SOD, CAT, GP-x which were reduced by a high-fat diet (152).

Another study showed that a flavon-3-ol, epicatechin (Table 3), supplementation reduced oxidative stress parameters in the intestine, giving rise to the possibility that antioxidant protection leads to the preservation of intestinal permeability (158). This is significant since HFD increases intestinal permeability, which can also lead to an increased inflammatory response (159).

Zhang et al. (157) observed troxerutin (Table 3) reducing oxidative stress, evidenced by an increase in the activity of antioxidant enzymes such as SOD1 and CAT and a reduction in ROS. These changes suggest that this flavonoid’s primary mechanism of action is a reduction of oxidative stress with subsequent repression of insulin resistance (IR).

### 4.2. Inflammation

Adipose tissue is directly related to the inflammatory process; excess adipose tissue leads to obesity and lipo-inflammation- a low-grade chronic inflammation process (160). Obesity can trigger inflammation through increased intestinal permeability, leading to increased circulating LPS content from the walls of the intestinal bacteria. The increase in LPS levels can trigger an inflammatory cascade (160). Flavonoids have been postulated to possess and exhibit anti-inflammatory properties attributed to their antioxidant mechanisms, inhibition of enzymes involved in eicosanoid synthesis or through the activation of gene expression of proinflammatory molecules- reducing the inflammatory process (161).

Several groups of flavonoids, such as cyanidin-3-glycoside, nobiletin, quercetin, troxerutin, and wogonin (Table 4), help mitigate obesity. These flavonoids promote the reduction of pro-inflammatory cytokines, which can worsen oxidative stress and reduce concentrations of pro-inflammatory cytokines (133).

**Table 4:** *In-vivo* effects of flavonoids on inflammation in various models of obese mice.

Flavonoids/mice used	Dietary source	Duration and dosage	Key molecular changes/mechanisms	References
Epigallocatechin gallate (ECGC) HFD/male SpragueDawley rats	Tea, strawberries, pears, and hazelnuts.	16 weeks, 3.2 g/kg	↓ CDC8, TRL4, TRAF6, IKKb, p-IKKb, p-NFkB, TNF-α, IL-6 in white adipose tissue.	(96)
Cyanidin-3-glycoside male C57BLKS/J-Leprdb/Leprdb mice	Black elderberries, rubus, and bilberries.	16 weeks, 1 g/ml	↓ IL-6, MCP-1, TNF-a in the liver. ↑ TFAM, NRF1, NRF2 in brown and white adipose tissue.	(149)
Eriodyctiol (ED) HFD/ male C57BL/6N mice	Lemons, oranges and grapes.	16 weeks, 0.005% (w/w)	↓ IFN-c, IL-1b, IL-6, IL-10 in the serum.	(162)
Naringenin HFD/ male C57B6/6J mice	Grapefruits, sour orange, tart cherries, tomatoes, and Greek oregano.	14 days, 100 mg/kg	↓ p-JNK, Mac-2, MCP-1 in white adipose tissue.	(163)
Naringenin HFHCD/ male mice	Grapefruits, sour orange, tart cherries, tomatoes, and Greek oregano.	12 weeks, 3% (w/w)	↓ CD68+ cells, CD45+, CD115-, GR-1+ cells, CD35+, CD115+, CD45+, CD115+, Lin-, Sca-1+	(150)
Silymarin HFD/male C57TBL/6J mice	Milk Thistle	30 days, 30 mg/kg	↓ p40-phox, p47-phox, p67-phox, NOX2, NOX4, NOXO1, NO, p47-phox, p67-phox, TRL4, iNOS, P65 nucleous. ↑ P65 cytoplasm and IkBa.	(151)
Baicalin HFD/ male SD rats	Fruits, root barks and leaves	16 weeks, 80 mg/kg	↓ TNF-α in the serum.	(164)
Morin HFD/male ICR mice	Figs, sweet chestnut, jack fruit, red wine, seaweed, tea, coffee, and cereal grains.	6 weeks, 50 mg/kg	↓ MCP-1 and MDA in serum. ↓ MDA, SOD and CAT in liver.	(153)
Morin HFD/male ICR mice	Figs, sweet chestnut, jack fruit, red wine, seaweed, tea, coffee, and cereal grains.	6 weeks, 100 mg/kg	↓ MCP1 and IL-6 in serum.	(153)
Nobiletin HFD/male C57BL/6J mice	Citrus fruits	16 weeks, 0.02 % (w/w)	↓ TRL2, TRL4, NF-kB, TNF-α in white adipose tissue. ↓ TNF-α mRNA in liver. ↓ IFN-γ, IL-6, IL-1β in plasma.	(144)
Quercetin WD/male C57TBL/6J mice	Apples, onions, parsley, sage, tea, and red wine.	16 weeks, 0.05 % (w/w)	↓ IL-6, TRL-4, p65 nuclear, TNF-a, IL-6, NLRP3, caspase 1, GRP78, CHOP. ↑p65 cytosolic.	(155)
Quercetin HFD/male ICR mice	Apples, onions, parsley, sage, tea, and red wine.	6 weeks, 20 mg/kg	↓TNF-a, CDC8, MCP-1 in serum. ↓ p-JNK/JNK, TRL4, ATF-6, XPB-1 in white adipose tissue.	(156)
Quercetin HFD/male C57BL/6J mice	Apples, onions, parsley, sage, tea, and red wine.	20 weeks, 0.05% (w/w)	↓ TNF-α in the plasma. ↓ TBARS in the liver. ↑ Gpx1 and CAT in the liver too.	(154)
Quercetin HFD/male C57BL/6J mice	Apples, onions, parsley, sage, tea, and red wine.	6 weeks, 20 mg/kg	↓TNF-a, CDC8, MCP-1 in serum. ↓ p-JNK/JNK, TRL4, ATF-6, XPB-1 in white adipose tissue.	(156)
Troxerutin HFD/male C57TBL/6J mice	Tea, coffee, cereal grains and vegetables.	20 weeks, 150 mg/kg	↓ p65 nuclear, p-PERK, p-eIF2, pIRE1, TRAF2, NOD1, NOD2, 4-HNE, IL-1β, TNF-α, MCP-1 in the liver. ↑p65 cytosolic, GSH, SOD1, CAT in the liver.	(157)
Myricetin HFD/male C57TBL/6J mice	Vegetables, fruits, nuts, berries, herbs, and tea, wine, fruit, and medicinal plants.	12 weeks, 0.12 % (w/w)	↑ Nuclear Nrf2, NQ01, HO-1, SOD2, CAT, GPx3, Prdx1, Prdx5 and TBARS in the liver. ↓ Cytosolic Nrf2 in the liver.	(152)
Wogonin HFD/male C57TBL/6J mice	Chinese Herbal Plants	2 weeks, 20 mg/kg	↓ TNF-α and IL-6	(165)
Genistein HFD + 10% SL/male SD	Soy-Based Foods	12 weeks, 0.01% (w/w)	↓TNF-α ↓ TRL4	(166)
Genistein HFD + 10% SL/male SD	Soy-Based Foods	12 weeks, 0.02% (w/w)	↓TNF-α in plasma. ↓ TRL4 in liver.	(166)

The upward arrow (↑) indicates an increase and the downward arrow (↓) indicates a decrease in the expression or activity. CD; Cluster of Differentiation: GLUT; Glucose Transporters; HFD: High Fat Diet; HFHCD: High-Fat-High-Cholesterol-Diet; IL: Interleukins; MCP: Monocyte chemoattractant protein; SOD: Superoxide Dismutase; TBARS: Thiobarbituric reactive substances; TNF: Tumor Necrosis Factor; WD: high-fat, high-cholesterol and high sucrose western style diet; (w/w): weight for weight.

Supplementation with the flavone eriodyctiol (ED; Table 4) reduces plasma leptin levels and levels of pro-inflammatory cytokines (IFN-c, IL-1b, IL-6) as well as increasing levels of IL-10, an anti-inflammatory cytokine, in the plasma (167). A separate study by Kwon & Choi showed that the flavanone eriocitrin could help reduce plasma levels of pro-inflammatory mediators and adipokines, suggesting that eriocitrin protects against the metabolic inflammation associated with obesity (162).

Another flavone, Baicalin (Table 4), was shown (164) to have a protective effect against liver steatosis (fatty liver disease) through the reduction of levels of a pro-inflammatory cytokine: TNF- α. Moreover, supplementation of the isoflavone genistein (Table 4) in mice fed an HFD showed that genistein reduced the progression of non-alcoholic fatty acid liver disease (NAFLD) and reduced inflammation by reducing TLR-4 activation and serum levels of TNF- α (164).

Morin (Table 4), a flavonoid, has also been shown to reduce the accumulation of hepatic triglyceride (TG) and attenuate the hepatic inflammation-associated lipid accumulation in HFD-fed mice (153). Supplementation with naringenin (Table 4) for early-phase HFD-induced obesity reduced the penetrance of Mac-2 and MCP-1 in adipose tissue; this effect is caused by inhibiting a responsible pathway for the expression of inflammatory cytokines (168). Silymarin (Table 4) decreased the relative expression level of TLR4, iNOS, and NO, which helps limit and activate the NF-kB signaling pathway and attenuate inflammation (151).

Toll-like receptors (TLRs) can identify patterns responsible for infection when active and can induce signaling pathways that stimulate inflammatory mediator expression and immune response (169). Epigallocatechin gallate (ECGC; Table 4) mitigates low-grade chronic inflammation by decreasing the expression of pro-inflammatory cytokines such as TNF- α and IL-6; it also suppresses TLRs, improving insulin signaling in adipose tissues (96). A separate study also provided evidence of EGCG’s ability to negatively regulate nitrosamine formation and proinflammatory markers.

Nobiletin (Table 4) supplementation improved hyperinsulinemia, insulin resistance, and glucose tolerance via decreased levels of circulating inflammatory cytokines and pro-inflammatory genes expression as well as TLR2 and TLR4 in adipose tissues (144). Another flavonoid, quercetin (Table 4), also showed similar activity through reducing JNK phosphorylation, TLR-4 activation, and the expression of pro-inflammatory mediators (155).

## 5. Anti-obesity effects of flavonoids through microbiota modification

The human body is home to numerous microorganisms, which mainly reside on the surface of the skin, gastrointestinal and respiratory tracts. These diverse communities of microorganisms are known as the microbiome (170). The use of high-throughput sequencing techniques linked to metagenomics has led to the discovery and detailed study of various unknown microbial communities linked to various life processes and ecosystems (171). Various research on the human microbiome has revealed the active role of gut microbiota in regulating major host functions like immune response, the circadian system, metabolism, and response to nutrition (170, 172). Variation in gut microbiota composition is also documented, and that can be attributed to host-microbiome dynamics and to various other factors, which include sex, age, genetic variations, and individual diet (171, 173).

Gut microbiotas enhance human’s proper digestion and absorption of food and its various components. They help decompose complex carbohydrates, proteins, vitamin biosynthesis, and absorption (170, 174, 175). Thus, gut microbiota changes can negatively impact human health and are related to several diseases (176). Among the different biochemical components of human food, flavonoids are essential to human health and can reduce the risk of various diseases, including cardiovascular disease. Interestingly, the human gut does not absorb most plant-based flavonoids (176, 177). However, these essential food components are metabolized by the gut microbiota, making them available for proper absorption in our gut. Interestingly, flavonoids act as prebiotics that can alter the diversity and profile of gut microbiota, which affect flavonoid metabolism and its absorption in the body (176, 177).

Flavonoids and gut microbiota can interact bidirectionally and thus affect human health. Firstly, foods rich in flavonoids can work like prebiotics and thus affect gut microbiota profile and function. Flavonoids can lower the levels of opportunistic species while positively affecting commensal and other helpful bacteria (178–180). Flavonoid metabolites can also suppress gut inflammation and help improve epithelial tissue function (178, 181).

Since flavonoids and gut microbiota interaction is a two-way process, gut microbiota can also affect the bioavailability and adsorption of the flavonoids. A minor percentage of dietary flavonoids are absorbed in our intestines without gut microbiota help. Instead, gut microbiota greatly affects the bioavailability and bioactivity of flavonoids after metabolizing them (177, 181). Notably, these phenolic metabolites can have synergistic or additive effects (182, 183). Probiotic supplementation in the long term could help bring gut microbiota that may enhance the bioavailability of flavonoids (181, 184). Indeed, several studies have shown the positive effect of anthocyanin-rich food in controlling obesity through interaction with gut microbiota (185, 186). Anthocyanins were described to control obesity through modulation of gut microbiota. These bioactive compounds are health-promoting anti-obesity prebiotics. Furthermore, anthocyanin-treatment attenuated overweight, hyperlipemia and improved hepatic lipid metabolism by regulating the expression of genes related to lipid metabolism in HFD obese mice (185, 186). Anthocyanins improved glucose metabolism and attenuated weight gain in diet-induced obese mice with intact gut microbiota (187). In fact, anthocyanins from various sources (purple corn, black soybean, blueberry, chokeberry, purple sweet potato, mulberry, cherry, grape or black currant) were demonstrated to modulate lipid metabolism and reduce fat mass in diet-induced obese rodent models. In a similar study, modulation of gut microbiota using flavonoids prevented obesity in a high-fat diet-induced obesity mouse model. Flavonoid extracts decreased the abundance of the bacterial family, Erysipelotrichaceae but did not affect the ratio of Firmicutes to Bacteroidetes (188).

The growth of gut microbiota is also dependent on the types of flavonoids. Studies have shown that the flavonoids (such as cyanidin-glycosylrutinoside and quercetin-rutinoside) from tart-cherries supported the growth of *Collinsella* and *Bacteroides* (189) and acted as prebiotics. Furthermore, flavonoids from Ougan juice (190) and mulberry leaves (191) were demonstrated to improve gut microbiota diversity, preventing obesity in high-fat diet-fed mice. Ougan juice flavonoids promoted short-chain fatty acid-producing bacteria (Blautia), *Lactobacillaceae, and Bacteroidetes while repressing Firmicutes and Erysipelatoclostridium. Mulberry flavonoid administration promoted acetic acid-producing bacteria and reduced adipose tissue weight in HFD-fed mice. Acetic acid production was mainly correlated to Bacteroidetes.*

Cranberry extracts are useful in controlling intestinal inflammation by supporting the *Akkermansia* spp. while inhibiting the *Bifidobacteria* (178). Similarly, the associated health benefit of green tea, rich in flavonoids, is attributed to a positive effect on *Bifidobacteria* spp. and *Lactobacilli* spp. growth (192). Quercetin can stimulate the growth of *Actinobacteria Proteobacteria* and *Firmicutes* (193). It also supports the growth of the probiotic *Lactobacillus rhamnosus* while inhibiting *Salmonella typhimurium*, a human gut pathogen (194). Another flavonoid, Baicalein, was shown to regulate the general profile of gut microbiota (195), whereas Kaempferol inhibited the growth of *Helicobacter pylori* (196). Studies have also shown the anti-bacterial effect of Anthocyanidin against human pathogens like *Enterococcus faecalis, Bacillus subtilis Pseudomonas aeruginosa and Staphylococcus aureus* (197).

Given their importance in regulating gut microbiota, flavonoids can be used as therapeutics. Restoring and maintaining gut microbiota using flavonoid supplementation to target health-beneficial microbiota could be a good approach.

## 6. Conclusion

The WHO has identified obesity as one of the leading health problems of the 21st century (1). It develops because of the energy intake/expenditure ratio imbalance, leading to excess nutrients and adipose tissue dysfunction (64). Flavonoids are one of the most significant bioactive substances among secondary metabolites, demonstrating remarkable anti-obesity properties (93). As evidenced by their capacity to reduce body weight, fat mass, and plasma triglycerides/cholesterol in both *in vitro* and *in vivo* models, the research strongly supports that most common flavonoids exhibit a considerable influence on obesity (92). The effects of flavonoids on obesity can be seen via various mechanisms, including decreased calorie intake and fat absorption, increased energy expenditure, altered lipid metabolism, increased inflammation and oxidation, and altered gut microbial profile. Flavonoids also help to restore the lost balance caused by the dysregulation of lipogenesis and lipolysis (92). They are involved in activating the AMP-activated protein kinase (AMPK), a key enzyme involved in controlling lipid metabolism and adipogenesis. They also activate the sympathetic nervous system (SNS), increase thermogenesis by promoting the production of adrenaline and thyroid hormones and induce white adipose tissue browning through the AMPK-PGC-1/Sirt1 and PPAR signaling pathways and are also believed to enhance the differentiation of brown preadipocytes, prevent apoptosis, and produce inflammatory factors in brown adipose tissue (BAT) (66). Therefore, it is anticipated that flavonoids could 1 day be used as anti-obesity drugs, given their demonstrated capacity to affect practically all the known obesogenic pathways. However, several aspects like bioavailability and dosage, off-target toxicity, adverse side effects, impact of gut microbiota on absorption, and use in combination therapy must be appropriately investigated before these molecules could be used to prevent or treat obesity in clinical settings.

## 7. Future perspectives

Although significant advances have been made in improving and clarifying the metabolism, bioavailability, and anti-obesity properties of flavonoids, assessing their full potential has nevertheless been challenging. Only a few flavonoids investigated for managing weight control directly contribute to weight loss by activating multiple mechanisms. The emerging aim for treating obesity is focusing on multiple pathways. Several studies have demonstrated that the bioavailability and safety of flavonoids changed when they were included in a food matrix (198). Although most of the assays have been done with *in vitro* models of digestion, it seems that the food matrices protect bioactive compounds from intestinal degradation. Understanding how dietary flavonoids interact with the food matrix will aid in developing food products with higher positive health impacts for the consumer (198). Furthermore, the absorption of flavonoids in the human body is dose- and type-dependent, affecting their bioavailability and pharmacokinetics. They show a low absorption rate and limited stability when they pass through the intestinal tract, where the microbiome may contribute to their absorption. Once absorbed, they enter the portal circulation, which brings structural changes in the molecules that alter their properties. Therefore, it is essential to consider how intestinal digestion and microbiota affect their uptake, metabolization, and bioavailability (199). Some studies also show that flavonoids enhance the production of glucagon-like-peptide (GLP-1) production, which positively affects gut bacteria (200). More thorough investigations are needed to determine this point because the gut microbiota significantly impacts the overall physicochemical and pharmacokinetic properties of flavonoids (201).

In addition to metabolism, membrane transporters, primarily efflux transporters, have long been known to influence drug absorption and bioavailability. Nevertheless, much remains about how cells handle flavonoids (202).

Pharmacokinetic studies have shown that the half-life of a typical flavonoid ranges from 1–2 h. The oral bioavailability of flavonoids is typically 10% or less (mainly in animals), with a range of 2-20% being relatively frequent. To improve the bioavailability of flavonoids, we must overcome several development hurdles, including solubility, permeability, metabolism, excretion, uptake, and disposition. Studies are needed to be performed to demonstrate how changes in the structure of flavonoids affect their solubility and dissolution rates and how various pharmaceutical excipients might be employed to increase dissolution rates. Methylation, acylation, glycosylation, and prenylation are common substitution patterns that afford diversity in the structure of flavonoids and modulate their properties. Glycosylation usually modifies the metabolism and absorption of flavonoids but fetters their bioavailability. Methylation could be used to improve bioavailability, but the lack of ability to improve bioavailability other than using methylated prodrugs could impede the use of flavonoids as drugs since methylation often adversely affects the activity. More bioavailable or extremely bioavailable formulations or derivatives are highly desirable since they will be less difficult to produce and test (203). The pharmacokinetic characteristics of flavonoids and other natural substances are significantly improved by nanosuspension technology, and there is much room for further research in this area (204).

Nanocarriers can potentially improve flavonoid bioavailability (205). Nanoparticles mainly include polymeric, solid-lipid nanoparticles, nanostructured lipid carriers, micelles, liposomes, nanosuspensions, and nanoemulsions. The pharmacokinetic parameters reveal that nanosuspension enhances absorption and increases the bioavailability of flavonoids (204). Encapsulation of flavonoids with appropriate materials that would improve their delivery, retention capacity, particle/food matrix interactions, and release profile would significantly improve the properties of flavonoids (206).

Flavonoid glycosides show improved stability and bioavailability, and the metabolic engineering technique is an efficient and promising way to glycosylate the flavonoids. The discovery of new glycosyltransferases and the establishment of an efficient metabolic network will be important for synthesizing specific flavonoid glycosides (207).

The complexation of flavonoids with cyclodextrins is a promising approach to improving their stability, aqueous solubility, rate of dissolution, and bioavailability (208).

A combination of flavonoids with known anti-obesity drugs or other natural products could be useful in treating obesity due to the inhibition of fat accumulation and the promotion of lipodieresis (209). Some studies demonstrate the beneficial effects of flavonoids in combination with procyanidin (20) by inducing satiety, satisfying hunger, or reducing craving urges. Still, little is known about the effect of combining different flavonoids or combining flavonoids with known drugs. It remains vastly unexplored whether they will have synergic, additive, or antagonistic effects.

The extent to which human diets must be tailored for maximum health is unknown, but a one-diet-suits-all appears implausible. On the other hand, personal or precision nutrition necessitates a greater focus on the peculiarities of nutrient-microbe interactions (210).

## Author contributions

AC, NM, and DB: conceptualization, supervision, project administration, and funding acquisition. AnM, SMS, ArM, MYW, AC, and NM: literature review and resources. AnM, SMS, ArM, MYW, SG, AC, and NM: writing—original draft preparation. AC, NM, SMS, and DB: writing—review and editing. SMS, AnM, and NM: figures and tables preparations and editing. AC: visualization. All authors contributed to the article and approved the submitted version.

## Funding

SMS was supported by a National Priorities Research Program grant (NPRP11S-1214-170101; June 2019-current) awarded to DB, from the Qatar National Research Fund (QNRF; https://mis.qgrants.org/Public/AwardDetails.aspx?ParamPid=fghmdaekde).

## Conflict of interest

The authors declare that the research was conducted in the absence of any commercial or financial relationships that could be construed as a potential conflict of interest.

## Publisher’s note

All claims expressed in this article are solely those of the authors and do not necessarily represent those of their affiliated organizations, or those of the publisher, the editors and the reviewers. Any product that may be evaluated in this article, or claim that may be made by its manufacturer, is not guaranteed or endorsed by the publisher.
